# Monitoring Bioaccumulation (in Gills and Muscle Tissues), Hematology, and Genotoxic Alteration in *Ctenopharyngodon idella* Exposed to Selected Heavy Metals

**DOI:** 10.1155/2020/6185231

**Published:** 2020-04-13

**Authors:** Nazish Shah, Ahsan Khan, Riaz Ali, Kasi Marimuthu, Muhammad Nazir Uddin, Muhammad Rizwan, Khaliq Ur Rahman, Mukhtar Alam, Muhammad Adnan, Muhammad, Shahibzada Muhammad Jawad, Saddam Hussain, Muhammad Khisroon

**Affiliations:** ^1^Department of Zoology, University of Peshawar, Peshawar, Khyber Pakhtunkhwa, Pakistan; ^2^Department of Zoology, University of Swabi, Anbar, Swabi, Khyber Pakhtunkhwa, Pakistan; ^3^Department of Biotechnology, Faculty of Applied Sciences, AIMST University, 08100 Bedong, Kedah Darul Aman, Malaysia; ^4^Center for Biotechnology and Microbiology, University of Swat, Swat, Khyber Pakhtunkhw, Pakistan; ^5^Department of Chemistry, University of Swabi, Anbar, Swabi, Khyber Pakhtunkhwa, Pakistan; ^6^Department of Agriculture, University of Swabi, Anbar, Swabi, Khyber Pakhtunkhwa, Pakistan; ^7^Department of Microbiology, University of Swabi, Anbar, Swabi, Khyber Pakhtunkhwa, Pakistan; ^8^Department of Zoology, Islamia College Peshawar, Peshawar, Khyber Pakhtunkhwa, Pakistan; ^9^Department of Plant Breeding and Genetics, University of Agriculture Peshawar, Peshawar, Khyber Pakhtunkhwa, Pakistan

## Abstract

Health and environmental problems arising from metals present in the aquatic ecosystem are very well known. The present study investigated toxicological effects of LC_15_ of metals such as copper, chromium, and lead for 24, 48, 72, and 96 h on hematological indices, RBC nucleus and cell morphology, and gill and muscle tissues of grass carp (*Ctenopharyngodon idella*). Experimental dose concentrations of copper were 1.5, 1.4, 1.2, and 1 mgL^−1^. Similarly, dose concentrations of chromium were 25.5, 22.5, 20, and 18 mgL^−1^ while those of lead were 250, 235, 225, and 216 mgL^−1^, respectively. Maximum decrease in the concentration of Hb, RBCs, and monocytes was observed against chromium, while maximum increase in the concentration of lymphocytes was reported against lead. Abnormalities such as single and double micronuclei, deformed nucleus, nuclear shift, irregular nucleus, deformed cells, microcyte cells, and vacuolated and swollen cells were observed. Gill tissues absorbed maximum concentration of lead followed by chromium and copper. Muscle tissues also absorbed maximum concentration of lead followed by chromium and copper, respectively. Histological alterations such as epithelial lifting, interlamellar spaces, club gill filaments, gill bridging, curling filaments, swelling and fusion of cells, irregular cells, destruction of epithelial cells, cellular necrosis, and inflammatory cells were observed in gill tissues while inflammation and necrosis of muscle fibers, degeneration of muscle fibers, edema of muscle bundles, zig-zag of muscle fibers, and lesions were observed in muscle tissues of fish exposed with different doses of these heavy metals, indicating the toxicity of metals to aquatic fauna as well as to human being via food chain.

## 1. Introduction

Environmental pollution is a worldwide problem across the globe and has adverse impacts on human health [1]. Over the last few decades, there is an increase in global concern over public health due to increase in environmental pollution [2]. Comparatively, human exposure to environmental pollutants is more intense nowadays to early days when life on earth came into existence [3]. Excessive concentrations of pollutants due to municipal wastes and burning of fossil fuels cause maximum damage to humans, animals, and plants including tropical rainforests, as well as the wider environment [4].

Metals as an important environmental pollutant refer to any metallic element that has a relatively high density and is toxic or poisonous even at low concentration [5]. Metals are widely distributed in aquatic bodies and are considered as essential in trace amount for normal biological activities of aquatic fauna [6]. Metals have gained much consideration among the nondegradable noxious and toxic substances [7]. Metals that accumulate in higher concentration cause harmful effects on the blood and organs of the aquatic organism by reacting with enzymes, deoxyribonucleic acid, ribonucleic acid, and cellular proteins [8]. Metal pollution of surface and underground water sources results in considerable soil pollution, and pollution increases when mined ores are dumped on the ground surface for manual dressing [9].

Copper is an essential element for the entire biota as lacking this nutrient can trigger enzymatic dysfunctions, but it can also be toxic to fish when present in high concentrations in the water [10]. Over the past two decades, the histopathological alterations in gills under acute and chronic exposure to metals have been studied in many fish species [11, 12]. Lead is the nonessential and most toxic metal which is widely distributed in the aquatic environment and earth's crust. Heavy metals such as lead, mercury, and cadmium are considered to cause public health hazards [13, 14].

The nonessential components of lead may cause nephrotoxicity, neurotoxicity, decrease growth rate, survival, metabolisms and development, and several adverse health effects [15, 16]. Chromium (Cr) in hexavalent form is comparatively active in the surrounding and is extremely toxic which may cause cancer and embryonic defects in aquatic organisms [17]. Cr(V1) compounds are very toxic even at low concentrations, but the toxicity depends on the pH value of the aquatic body [18]. Yousafzai [19] reported high concentration of Cr in the gills of *Labeo dyocheilus* and *Wallago attu*. The chromium absorption in canals and seas is specified to range from 1 to 10 *μ*gL^−1^ and EPA approval for the acceptable side by side are 50 to 100 *μ*gCrL^−1^ for the safety of human healthiness and water life individually [5].

The excessive uptake of essential and nonessential metals ends in accumulation in various tissues [20]. Metals in higher concentration change the biological activities of the fish [20]. Consumption of such metal-contaminated fishes by a human can cause serious health issue [21]. Metals deteriorate the ecological balance of the aquatic environment [22, 23] because fish are at the end of the aquatic tropic level and they have a higher tendency to accumulate metals in their body [23]. In the aquatic system, they diffuse radially and fish often being on the top of the aquatic food chain are more susceptible to the hazardous effects as compared to terrestrial vertebrates and it is critical to investigate and monitor the bioaccumulation pattern [24, 25].

The aim of the present study was to investigate heavy metal bioaccumulation and alteration in hematological indices and red blood cell and nucleus morphology and in different organs like gills and muscles of grass carp exposed with different concentrations of heavy metals.

## 2. Materials and Methods

### 2.1. Animal

Grass carp (*Ctenopharyngodon idella*: 8.5 ± 5.5 cm; 9.5 ± 6.5 g) were transported in oxygenated bags (50 fish per bag) from carp hatchery of Mardan and Peshawar to the lab. The fish were treated with 0.2% KMnO_4_ solution for two minutes to remove any external infection. All the fish specimens were then acclimatized for two weeks in aquarium having tap water. After acclimatization, the fish were moved to experimental aquariums for experimentation. In both acclimatization and experimentation aquariums, fish were fed with commercial carp pellet diet of Oryza Organics, Pakistan (composed of protein = 20.0%, fats = 3.0%, fiber = 7.0%, moisture = 10.0%, calcium = 0.7%, and phosphorus = 0.7%) on each alternate day. Physiochemical parameters of water were also recorded on every alternate day during acclimation and exposure period and were found in permissible limits as per the recommended values of APHA and American Public Health.

### 2.2. Experimental Design

After acclimatization, fishes were divided into three groups (groups I, II, and III) of 10 fish per group and exposed to LC_15_ of heavy metals such as copper, lead, and chromium for 24, 48, 72, and 96 hours, respectively. The experiment was conducted in semistatic conditions, following OECD Guideline Number 203 [26]. LC_15_ of copper was 1.5, 1.4, 1.2, and 1 mgL^−1^. Similarly, LC_15_ of lead was 250, 235, 225, and 216 mgL^−1^ while LC_15_ of chromium was 25.5, 22.5, 20, and 18 mgL^−1^, respectively. After the stipulated time, three fish were randomly selected and anesthetized using clove oil [27]. The anesthetic was prepared fresh by dissolving clove oil into absolute alcohol (Merck, Germany) in a ratio of 1 : 2. Fish were dissected manually, and the required tissues were removed, weighted, and were stored at −20°C for further analysis.

### 2.3. Hematological Parameters

For the examination of the hematological parameter, blood was drawn from the caudal vein just behind the anal pore of grass carp through a sterile heparinized syringe (5 cc) having half the size of the needle. After collection, the blood samples were transferred to ethylenediaminetetraacetic acid (EDTA) tubes to prevent blood clotting. The blood was gently shaken in EDTA tubes so that the blood gets mixed with the anticoagulation drops in the EDTA tube to prevent blood coagulation, and then, the blood samples were observed through hematological analyzer (Model no. URIT 3020).

### 2.4. RBC Cell and Nucleus Morphology Analysis

A thin smear of blood from each treatment was made on precleaned slide and fixed in methanol for 20 min after drying. The slides were air-dried and stained with Giemsa staining (6%) for 25 min, washed with tap water, allowed to dried, and examined at 100x magnification under a microscope (Nikon with DS-L3 camera) [28]. Cell and nuclear abnormalities were calculated according to the method proposed in [29] as mentioned below:

(1) $$\begin{array}{c} \text{Cell and nucleus abnormalities}=\frac{\text{number of abnormal cells and nucleus}}{\text{total number of cells and nucleus scored}}\times 100. \end{array}$$

### 2.5. Tissue Digestion for Accumulation

Estimation of heavy metals was carried out by following the tissue digestion. Tissue samples were thawed, rinsed in distilled water, and blotted with blotting paper. After blotting, the samples were transferred to 100 ml volumetric flasks. The entire flask was washed properly and rinsed with distilled water, before transferring the tissue samples. Then, the known weights of each tissue were transferred to these volumetric flasks. Samples digestion was carried out according to the methods presented in [30, 31]. A slight modification was made in the procedure [32]; instead of putting 10 ml nitric acid (60%) and 5 ml per chloric acid (70%) at the time of digestion, 5 ml nitric acid (60%) and 1 ml per chloric acid (70%) were added to each flask and the flasks were then kept overnight.

The next day, a second dose of 5 ml nitric acid (60%) and 4 ml per chloric acid (70%) was added to each flask. The flasks were kept on a hot plate, covered with Pyrex glass cover, and allowed to digest at 200 to 250°C until a clear transparent solution was observed. Initially, dark brown fumes appeared followed by white fumes. The dense white fumes from the flask, after brown fumes, were an intimation of completion of the digestion process. By this method, digestion was accomplished in about 30 minutes instead of 3 to 4 hours as described in [28]. After digestion, the samples were cooled, filtered through Whatman 42 filter paper and diluted to 100 ml with distilled water by proper rinsing of the digestion beakers. Samples were stored in properly washed glass bottles until the metal concentration determination. Determination of heavy metals was carried out through atomic absorption spectrophotometer, Agriculture University, Peshawar (Model no. Spectra AA 2000).

### 2.6. Histological Studies

After the fish dissection, portions of tissues (gills and muscles) were preserved in 10% formalin for histological studies. The preserved tissues were processed in various grades of ethanol, cleared in xylene, and impregnated with wax (mp; 58°C). Five-micron-thick sections were cut using a rotary microtome (Leica RM 2165) at 100x. Tissue sections were stained with hematoxylin and eosin (H&E). Stained slides were observed and photographed under a high-resolution microscope (Lecia, Japan) fitted with a digital camera [33].

### 2.7. Statistical Analysis

The statistical analysis was performed using IBM SPSS (version 20). All data were expressed as mean ± SD.

## 3. Results

Maximum decrease in the concentration of hemoglobin against copper doses was noticed at exposure time of 72 h in comparison with the control group. Similarly, the concentration of RBCs and monocytes also decreased against copper at exposure times of 72 and 96 h, respectively, while the lymphocyte concentration increased against various doses of copper and the maximum increase was noticed against 24 and 96 h exposure, respectively, as shown in Table 1.

Hemoglobin and RBCs showed a decline in their concentration against chromium and maximum decreases were observed against the exposure time of 96 and 24 h, respectively, as shown in Table 2 while decrease in monocyte concentration was also noticed against each exposure time except for 24 h. Lymphocyte showed an increase in the concentration against various doses of chromium except for 24 h exposure as shown in Table 2.

Lead toxicity declines the concentration of hemoglobin and the maximum decline was reported against 96 h exposure. Similarly, RBCs and monocyte also end with decreases in their concentration against various doses of lead, but the maximum decrease was noticed against 72h and 96 h exposure, respectively, while lymphocyte against the toxic media of lead ends with increase in concentration and the maximum increase was noticed at 96 h exposure as shown in Table 3.

### 3.1. Bioaccumulation

During 24 h exposure, maximum concentration of lead was accumulated in gill tissue that was 4.113 ± 0.831 mg/g followed by chromium 0.537 ± 0.250 mg/g and copper 0.266 ± 0.029 mg/g. Similarly, in muscle tissue, lead was accumulated in maximum concentration that was 6.697 ± 1.475 mg/g followed by chromium 0.593 ± 0.393 mg/g and copper 0.038 ± 0.016 mg/g, respectively, as shown in Table 4. Likewise, treatment of fish for 48 h indicated the maximum accumulation of lead in gill tissue that was 1.037 ± 0.418 mg/g followed by chromium 0.240 ± 0.130 mg/g and copper 0.048 ± 0.038 mg/g. Similarly, in muscle tissue, lead was accumulated in maximum concentration that was 3.147 ± 1.606 mg/g followed by chromium 0.847 ± 0.257 mg/g and copper 0.047 ± 0.054 mg/g, respectively, as shown in Table 4. After the exposure of fish for 72 h, gill tissue absorbed lead in maximum concentration that was 1.647 ± 0.136 mg/g followed by chromium 0.667 ± 0.331 mg/g and copper; 0.024 ± 0.007 mg/g. Similarly, in muscle tissue, the lead was accumulated in maximum concentration that was 3.253 ± 0.992 mg/g followed by chromium 0.477 ± 0.162 mg/g and copper 0.021 ± 0.002 mg/g, respectively, as shown in Table 4. Treatment of fish for 96 h indicated the maximum accumulation of lead in gills tissue that was 3.720 ± 3.008 mg/g followed by chromium 0.937 ± 0.718 mg/g and copper 0.036 ± 0.019 mg/g. Likewise, in muscle tissue, lead was accumulated in maximum concentration that was 3.730 ± 1.267 mg/g followed by chromium 0.307 ± 0.257 mg/g and copper 0.013 ± 0.007 mg/g, respectively, as shown in Table 4. Therefore, the present study indicated that gill and muscle tissues showed maximum affiliation towards lead accumulation followed by chromium and copper.

### 3.2. RBC Cell and Nucleus Morphology

**Figure 1:**
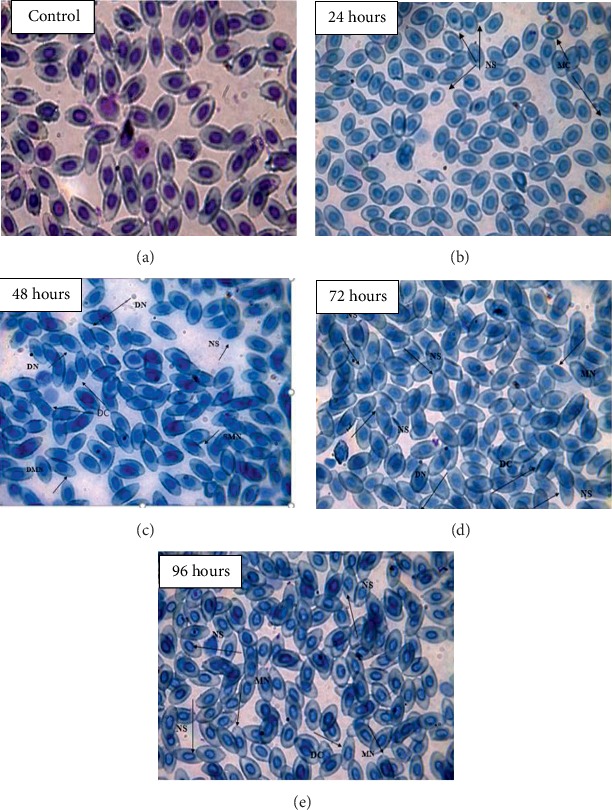
(100x) Showing RBC nucleus and cell abnormalities in comparison with the control group against the treatment LC_15_ of copper: (a) control; (b) 24 hours; (c) 48 hours; (d) 72 hours; (e) 96 hours.

Slides were also analyzed after exposing the fish to copper for different time periods. The RBC nucleus abnormalities and other erythrocyte alterations in *C. idella* were calculated and compared with the control group. Against copper toxicity in nuclear abnormalities, maximum micronuclei were observed at 24 and 96 h exposure, double micronuclei were maximum at 48 and 72 h, maximum deformed cell and nuclear shift were observed at 96 h, lobed nucleus was maximum at 72 h, and irregular nucleus was maximum at 96 h exposure while in cellular abnormalities, deformed cells, microcytes, and vacuolated and swollen cells were maximum at 48 and 24 h exposure, respectively, as shown in Table 5 and Figure 1.

**Figure 2:**
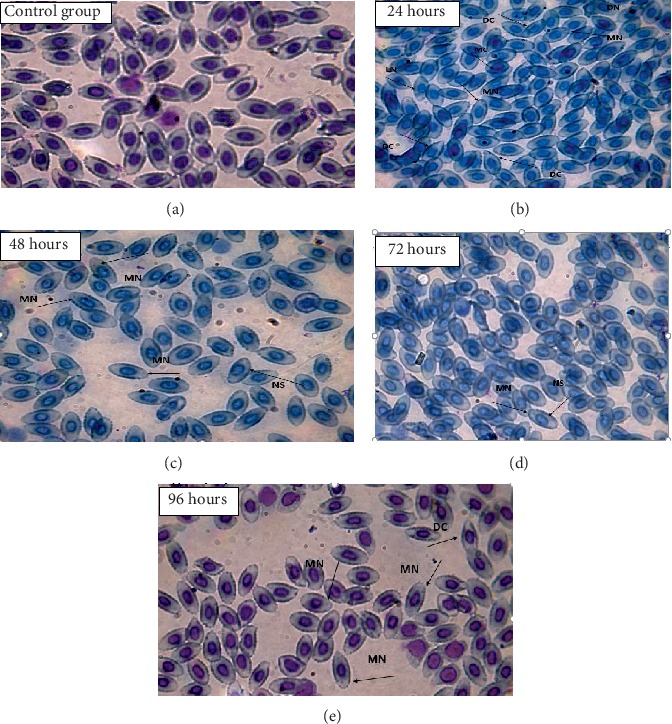
(100x) Showing RBC nucleus and cell abnormalities in comparison with the control group against the treatment LC_15_ of lead: (a) control group; (b) 24 hours; (c) 48 hours; (d) 72 hours; (e) 96 hours.

Similarly, lead intoxication ends in maximum single micronucleus at 96 h while no double micronuclei were observed. Maximum deformed nucleus was observed at 72 h, nuclear shift at 48 h, and lobed nucleus at 24, 48, and 72 h, and maximum irregular cells were observed at 96 h, respectively. In cellular abnormalities, deformed cells and microcytes were maximum at 72 h and vacuolated cells and swollen cells were maximum at 48 h, respectively, as shown in Table 5 and Figure 2.

**Figure 3:**
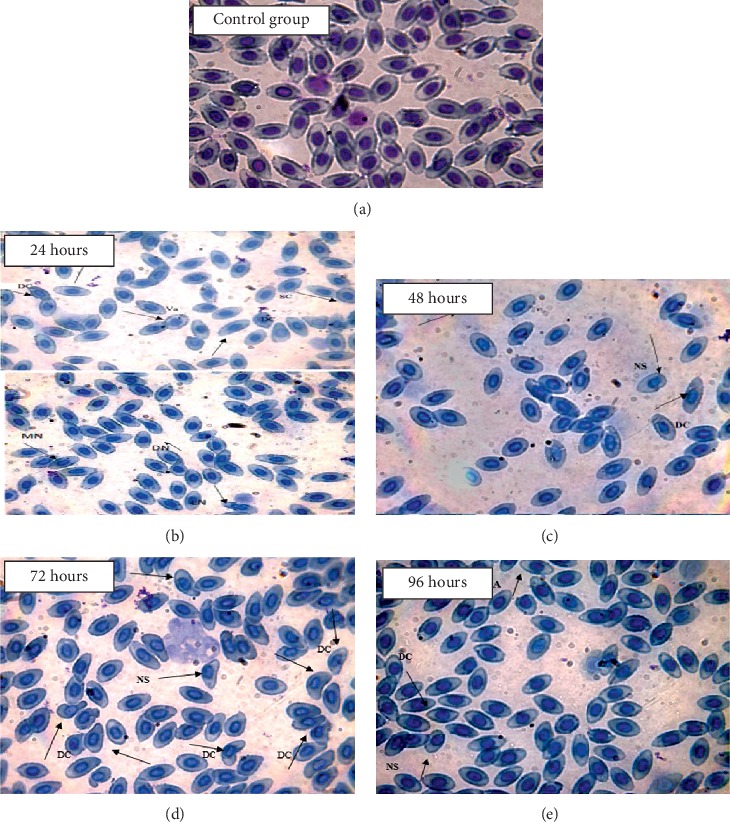
(100x) Showing RBC nucleus and cell abnormalities in comparison with the control group against the treatment LC_15_ of chromium: (a) control group; (b) 24 hours; (c) 48 hours; (d) 72 hours; (e) 96 hours.

Against chromium toxicity, maximum single micronuclei were observed at 48, 72, and 96 h while no double micronuclei were noticed. Similarly, maximum deformed nucleus was observed at 48 h, nuclear shift at 72 h, and lobed nucleus at 24 and 96 h, and maximum irregular cells were observed at 48 h exposure, respectively. In cellular abnormalities, maximum deformed cells were observed at 72 h, microcytes at 48 h, and vacuolated and swollen cells at 96 h exposure, respectively, as shown in Table 5 and Figure 3.

### 3.3. Histopathology

**Figure 4:**
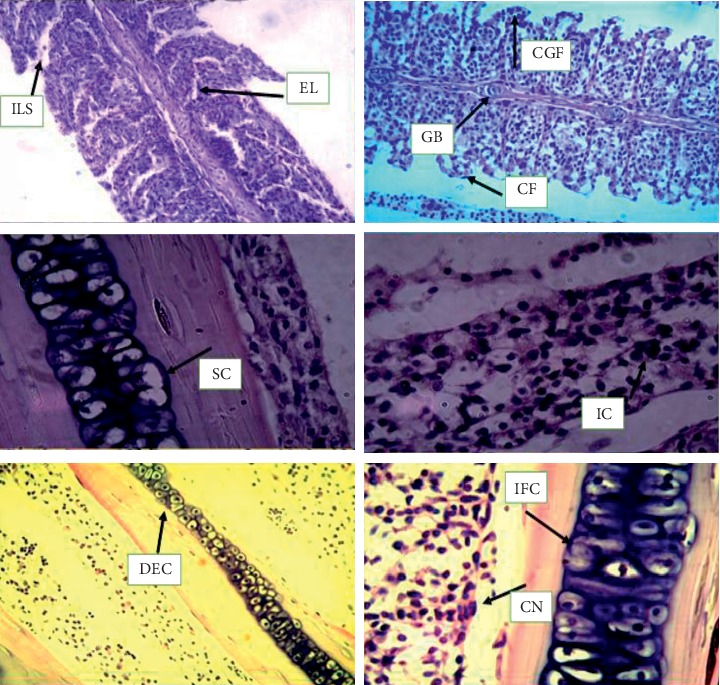
5-micron-thin gill section of *Ctenopharyngodon idella* (hematoxylin and eosin stain) (H&E) (100x) representing various alterations in gill tissues against copper, chromium, and lead as interlamellar spaces (ILS), epithelial lifting (EL), gill bridging (GB), club gill filaments (CGF), curling filaments (CF), swell cells (SC), irregular cells (IC), destructive epithelial cells (DEC), cellular necrosis (CN), and inflammatory cells (IFC).

**Figure 5:**
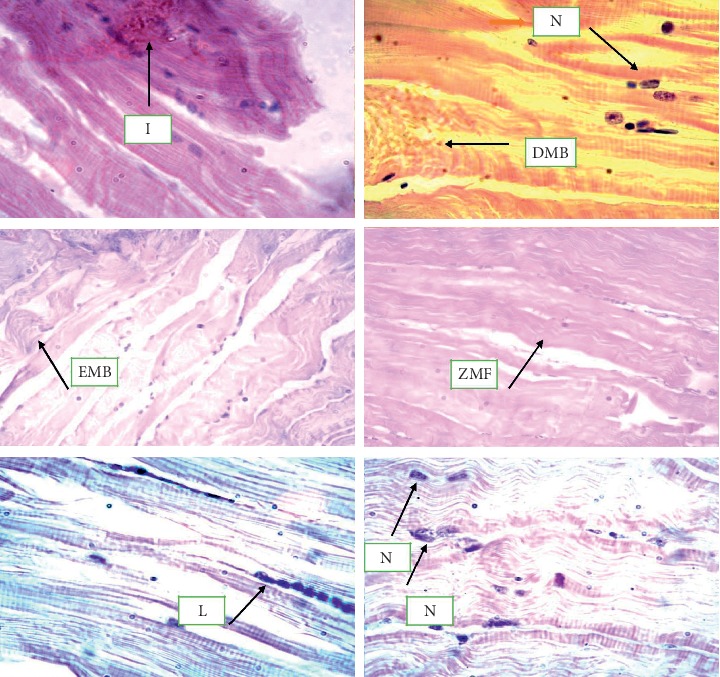
5-micron-thin muscle section of *Ctenopharyngodon idella* (hematoxylin and eosin stain) (H&E) (100x) representing various alterations in gill tissues against copper, chromium, and lead inflammation (I) and necrosis of muscle fibers (N), degeneration of muscle fibers (DMB), edema of muscle bundles (EMB), zig-zag of muscle fibers (ZMF), and lesion of muscle tissues (L).

Various pathological alterations against copper, chromium, and lead toxicity have been observed as shown in Figures 4 and 5.

## 4. Discussion

Bioaccumulation of heavy metals is used for environmental monitoring largely because aquatic organisms are in direct contact with the contaminated water. Tissue metal concentrations in fish are good indicators of aquatic system exposure to metal contamination [34, 35]. Heavy metals accumulate in fishes via water, sediments, and food such as algae upon which both herbivorous and omnivorous fishes feed [6, 36, 37]. Difference in the absorption of heavy metals depends on the heavy metal affiliation, the fish species, and the physical and chemical properties of water [24]. The heavy metals are accumulated in living organisms when they are taken up and stored faster than they are broken down (metabolized) or excreted. They enter into the water supply by industrial and consumer materials, or even from acidic rain breaking down soils and releasing heavy metals into streams, lakes, rivers, and groundwater. The three important environmental heavy metals such as Cu, Pb, and Cr have been reported, but some other heavy metals can also badly affect the environment. Heavy metal toxicity has been reported to be caused by different means, e.g., from contamination of drinking water (Pb pipes), high ambient air concentrations near emission sources, or from the food chain.

The current study was conducted which aimed at analyzing the accumulative concentration of heavy metals such as copper, lead, and chromium and to scrutinizing the effect of such accumulation on hematological parameters, RBC cell and nucleus morphology, and gill and muscle tissues of grass carp after exposing the fish for 24, 48, 72, and 96 h against LC_15_ concentration of each heavy metal for specific time periods. Decline in concentration of Hb, RBCs, and monocytes was observed and maximum decrease was noticed against chromium, copper, and lead, respectively, while maximum increase in concentration was reported in lymphocytes against lead. Abnormalities such as single and double micronuclei, deformed nucleus, nuclear shift, irregular nucleus, deformed cells, microcytes, and vacuolated and swollen cells were also observed against various doses. In the present study, maximum concentration of lead was accumulated followed by chromium and copper while the toxicity level of copper was maximum followed by chromium and lead. In addition, the present study explores the toxicological effects of these metals on tissues particularly gills and muscles. Several abnormalities in gill and muscle tissues were observed.

Shah [38] has documented that exposure to heavy metals is the main root cause for reduction in the erythrocyte count and hemoglobin concentrations. Our results were also in the agreement with [39] which demonstrated that decrease in erythrocyte count and hemoglobin concentration may perhaps be due to the blocking of genes in bone marrow or can be due to impairment in intestinal membrane to absorb enough amount of iron or may be hypoxic conditions and destruction of hematopoiesis that have been induced by exposure of specimen to selected toxicants.

The sublethal concentration of copper has significantly reduced erythrocytes, hemoglobin contents, and hematocrit values in *Channa punctatus*. Under copper-induced stress, anemia may occur due to the disordered synthesis of hemoglobin and also due to injury of blood cells [40, 41]. Our findings were also in correlation with findings of Mazon et al. [42] who observed that 3.2 mgL^−1^ of copper exposure to catfish (*Clarias lazera*) has resulted in anemia and hemolysis. Similar results were also documented according to [43] that sublethal exposure of 0.36 mgL^−1^ of copper for 45 days to *Channa punctatus* indicated a significant decline in RBCs, PCV, and hemoglobin concentrations when compared to the control group. Leukocyte concentration increased against severe physical stress and any infection of body tissues. Sampath et al. and Soiveo et al. [44, 45] have documented that leukocytes increased significantly when fish exposed to higher concentration of copper which is in correlation with the present findings.

Decline in erythrocyte and hemoglobin concentration was observed against lead in comparison with the control group in the present findings which is in correlation with [46, 47] that sublethal concentrations of lead, zinc, and copper exposure have resulted in hemolytic anemia due to break down of red blood cells along with reduction in red blood cells PCV% and Hb%. Similar result was also documented in [48] that, during stress condition, releasing of epinephrine, which might cause deformed red blood cells or premature release red blood cells, possibly disturbs the hematocrit values.

Mikryakov et al. [49] reported that 0.3 mgL^−1^ of lead ended in lymphocytosis and neutropenia in eel (*Anguilla anguilla*). Vinodhini and Narayanan [50] evaluated that due to lead intoxication, biochemical, hematological, and immunological damage has altered the growth of *Tilapia zilli*, even at very low concentration of heavy metals. Heavy metals Pb, Cu, Cr, Ni, Fe, Cu, Mn, Zn, Cd, and Hg induce alteration in morphology, physiology, biochemical parameter, and immune system [51].

Similarly, the present study reported that chromium intoxication resulted in a significant decline in the concentration of hemoglobin content and red blood cell counts at various times of exposure in comparison with the control group. Similar results were reported in [52] that decrease in hemoglobin content occurs due to chromium and causes alteration in synthesis of hemoglobin. Deficiency in hemoglobin and erythrocyte count of all exposed fish may be due to hemolysis or may be due to inhibition of enzymes necessary for hemoglobin synthesis. The present study showed similarities to the study of Gad [53] who reported that Cr VI exposure to *Cyprinus carpio* revealed a significant decrease in erythrocytes and hemoglobin. Similar result was also reported in [54] that 39.4 mgL^−1^ of chromium (VI) exposure displayed a significant decline in total erythrocytes count and hemoglobin after 24 and 96 h in *Labeo rohita*.

Monocytosis occurs due to lesions including extensive destruction of tissues. Monocytes have an important role in phagocytosis and also in ingestion of large particle such as necrotic cellular debris, large microorganism, and senescent cells as well as effective against the toxicant environment [55]. Similarly, in the present study, chromium exposure caused maximum increase in monocytes at 24 h to cope with the stress environment. Increase in lymphocyte counts against chromium at 96 h was observed in the present study which is in correlation with the results documented by Witeska et al. [56] who reported that fish species are susceptible to the deleterious effects of heavy metals as reflected in the blood changes such as lymphocytosis, anemia, and eosinophilia.

The present finding revealed that chromium exposure caused different abnormalities in the erythrocyte cell such as enlarged cell, irregular cell membrane, and ruptured cell membrane and also caused erythrocyte nucleus abnormalities including micronucleus, lobed nucleus, and irregular nucleus in *Ctenopharyngodon idella*. The present study documented various red blood cells, and nucleus abnormalities including single and double micronuclei, deformed nucleus, nuclear shift, irregular nucleus, deformed cells, microcytes, and vacuolated and swollen cells were observed against various doses. Similar finding was documented by Ergene et al. [29] who observed that LC_50_ of copper, lead, and cadmium exposure caused a significant increase in frequency of micronuclei and other nuclear abnormalities such as lobed cells, binucleated cells and notched in the erythrocytes of *Oreochromis niloticus*, *Poronotus triacanthus*, and *Puntius altus*. Erdoğrul and Erbilir [57] have documented that lead caused structural deformations, morphological changes in erythrocyte nucleus, and spreading of chromatin material. Similarly, the present finding was in correlation with the study of Dural et al. [58] who have observed a significant increase in the frequency of micronucleus and other nuclear abnormalities in erythrocytes of catfish and mullet against copper, lead, nickel, and cadmium exposure.

Gills are the first target of waterborne pollutants and heavily prone to accumulation of heavy metals due to the constant contact with the external environment and the main place for the uptake of heavy metals [59]. The heavy metal accumulation in gill tissues is due to absorption of heavy metals through the gill surface which is frequently difficult to eliminate them. The extremely branched morphology of gill tissues and the movement of water through it result in maximum accumulation of heavy metal [59]. The heavy metals do not contact directly with the muscle tissues, as compared to gill tissues which are completely exposed to an aquatic environment which correlates with the present study [60]. Intake of pesticides and toxic metals via gills ends in accumulation of these toxic chemicals in gills, thus damaging the gills' lamella which inturn affects the ion exchange mechanism during osmoregulation [61]. The gill tissues not only do respiration, but they also do osmoregulation [62]. Therefore, gill tissues as near to outer aquatic environment are affected directly by toxic chemicals, thus altering osmoregulation which justifies the present findings [63].

It has been reported that the accumulation of heavy metals in gills is because of its thinnest epithelium among all the organs of the body through which metals can easily pass [64]. Mastan [23] has also reported the similar pattern of bioaccumulation in *Labeo rohita*. Bioaccumulation of heavy metals like mercury, chromium, nickel, copper, and lead has been documented to be influenced by variation in age, season, and gender, and this may correlate with feeding habits in different seasons and areas [64–66]. The connection between feeding habits, foraging behavior, and heavy metal concentration is also well established as higher for omnivorous and herbivorous as compared to carnivorous. Feeding on different food chains is considered to have greater chances of heavy metal bioaccumulation [66]. Thus, the herbivore nature of grass carp makes it more prone to heavy metal bioaccumulation as compared to carnivore's fish.

It has been a common trend in most cases that accumulation is highest in gills and lowest in muscles. Gills, skin, and alimentary canal are the entry points of heavy metals. Active interaction of the tissue type, that is, gills with contaminated water and liver exposure to contaminated food is another reason for the concentration of heavy metals in the respective tissues [66].

It has been reported that that heavy metals accumulated maximum in the gills of *Labeo dyocheilus* and *Wallago attu* [31, 66]. In addition to it, other studies have also reported that higher concentration of heavy metals is a usual trend of bioaccumulation in different fishes mostly in gills such as *Channa punctatus* [66] and *Wallago attu* [67], while experimental studies have shown that muscles of *Labeo rohita* absorbed the least metal as compared to other organs [68].

The impact of heavy metals on gill tissues affects the function of the different enzymatic activities of carp and irreversible damage to the respiratory organs of fish murrel (*Channa striata*) [69]. In gill and muscle tissues of fishes, all the heavy metals absorb in different quantities according to the availability of heavy metals [70]. The gill tissue is exposed to the aquatic body, so its structure and function get altered maximum against the heavy metal concentration, and thus, the gill destruction occurs which ends in osmoregulation and respiratory function impairment, decreases the whirling motion of the fish, decreases the oxygen level, and finally causes death. The addition of toxic substances caused biological dysfunction or dysfunction of afferent parts of the body of fish [71].

Copper is an important metal in fish and is regulated in the muscle tissue with high-molecular-weight proteins (metallothionein-like). The copper concentration in muscle tissue showed a variation from 2.12 *μ*gg^−1^ to 27.94 *μ*gg^−1^ and average value of 7.54 ± 0.94 *μ*gg^−1^ for fishes from East Kolkata Wetlands. The maximum concentration was observed for *Hypophthalmichthys molitrix* (21.1 ± 6.1 *μ*gg^−1^) and minimum for *Puntius ticto* (2.6 ± 0.5 *μ*gg^−1^) [12].

The highest concentration of lead accumulation was found in the liver as compared to other tissues, followed by gill and muscle tissues of *Tilapia*, after exposing to the sublethal concentration of lead for time intervals. These findings recorded that liver is the prime site of metal binding in freshwater fishes. Lead accumulation in *Anabas testudineus* showed usual differences with a high degree of organ specificity after 30 days of exposure to a sublethal concentration of lead [72].

Other researchers reported heavy metal accumulation in tissues of *Tilapia*, a freshwater fish [53]. It was noted that Cr accumulation in the tissues followed sequence gills > skin > muscle tissues (least). Other studies found that the lowest concentration of Cr was detected in the muscle, skin, and gill tissues [73]. Another study indicated that concentrations of heavy metals on wild fish were higher in skin samples than in the muscle tissues [74].

The heavy metal damage is an important factor in many pathological and toxicological processes [75]. Gill is an important tissue because of its direct contact with water and any effect or agency has to go through it to come into the fish body. The lamella epithelial lining reacts with dissolved lead creating tissue osmoregulatory imbalance. The observed changes in gills such as hyperplasia, lamellar fusion, epithelial necrosis, and edema were generally attributed to the toxic effects of lead. Similar alterations in the gills have also been reported in the fish exposed to metals [76]. The histology of the gill has been shown to reflect different environmental conditions for the fishes and to be sensitive to copper exposure [76]. The study conducted by Ayoola and Alajabo [76] showed that marked histopathological changes have been found in the gill and liver of fish *Labeo rohita* under sublethal concentrations of chromium in chronic exposure. Fusion and shortening lamellae, hypertrophy, degeneration of epithelium, and necrosis were found in the gills of chromium-treated *Labeo rohita*. A higher degree of hypertrophy and fusion of gill lamellae were prominent in the gills of fish exposed for 30 days.

According to Nath and Banerjee [43], in muscle tissues, different histological alterations such as necrosis of muscle fibers, swelling, degeneration of muscle fibers, edema of muscle fibers, enlarged lesions in the epidermis of muscle tissue, inflammation, and zig-zag of muscle fibers were noticed at 7 days against 6.83 ppm concentration of copper. After 28 days of exposure in the lowest concentration of copper, the muscle tissues exhibited dystrophic changes with marked thickening and separation of muscle bundles. Significant changes noted are broken myofibrils and gap formation between muscle bundles, which finally led to degeneration in muscle bundles accompanied by focal areas of necrosis as well as atrophy in king mackerel, *Scomberomorus*.

Ayoola and Alajabo [76] have observed mild lesion, necrosis, inclusion of bodies, inflammation, and cellular degeneration in the muscle tissue of the fish *Oreochromis niloticus* exposed to aqueous and ethanolic extracts of *Ipomoea aquatica* leaf. Ayoola and Alajabo [76] studied the histopathology of fish *Cyprinus carpio* exposed to sublethal concentrations of lead and cadmium. The fish showed marked thickening and separation of muscle bundles with severe intracellular edema [37, 77]. The destruction and vacuolation of the muscle cells in *Oreochromis* spp. were observed in fish exposed to chromium [37, 77].

## 5. Conclusion

Toxicity of copper, chromium, and lead was reported against grass carp (*Ctenopharyngodon idella*) by scrutinizing hematological indices, RBC nucleus and cell morphology, and gill and muscle tissues. Decline in concentration of Hb, RBCs, and monocytes was observed and maximum decrease was noticed against chromium followed by copper and lead, respectively, while maximum increase in concentration was reported in lymphocytes against lead. Abnormalities such as single and double micronuclei, deformed nucleus, nuclear shift, irregular nucleus, deformed cells, microcytes, and vacuolated and swollen cells were also observed against various doses. Gill tissues absorbed maximum concentration of lead followed by chromium and copper. Muscle tissues also absorbed maximum concentration of lead followed by chromium and copper, respectively. Histological alterations such as epithelial lifting, interlamellar spaces, club gill filaments, gill bridging, curling filaments, swelling and fusion of cells, irregular cells, destruction of epithelial cells, cellular necrosis, and inflammatory cells were observed in gill tissues while inflammation and necrosis of muscle fibers, degeneration of muscle fibers, edema of muscle bundles, zig-zag of muscle fibers, and lesions, were observed in muscle tissues of fish exposed with different doses of these heavy metals, indicating the toxicity of metals to aquatic fauna as well as to human being via food chain.

## Figures and Tables

**Table 1:** Hematological indices in the blood of *C. idella* exposed to LC_15_ of copper for 24, 48, 72, and 96 hours.

Copper-exposed fish groups	Control				24 h LC_15_				48 h LC_15_				72 h LC_15_				96 h LC_15_			
	Fish I	Fish II	Fish III	Mean ± SD	Fish I	Fish II	Fish III	Mean ± SD	Fish I	Fish II	Fish III	Mean ± SD	Fish I	Fish II	Fish III	Mean ± SD	Fish I	Fish II	Fish III	Mean ± SD
Hemoglobin	16.1	12.6	12.2	13.6 ± 2.145	9.4	9.6	10.4	9.8 ± 0.529	9.1	5.2	10.1	8.1 ± 2.589	6.1	5.9	7.5	6.6 ± 1.101	6.3	8.5	8.1	7.6 ± 1.171
RBCs	4.05	3.16	3.07	3.4 ± 0.541	2.21	2.29	2.54	2.34 ± 0.0211	2.21	2.27	2.51	2.33 ± 0.0158	1.24	1.28	1.31	1.27 ± 0.035	2.23	2.23	1.94	2.15 ± 0.167
Lymphocytes	90.4	94.4	96.4	93.7 ± 3.054	98.8	96.9	96.6	97.4 ± 1.192	95.5	91.9	93.0	93.4 ± 1.844	85.5	93.9	83.7	87.6 ± 5.507	96.3	97.8	98.2	97.4 ± 1.002
Monocytes	8.3	4.4	3.0	5.2 ± 2.746	0.9	2.7	2.6	2.06 ± 1.011	3.8	7.5	5.8	5.7 ± 1.852	4.1	4.0	4.0	4.0 ± 0.577	2.9	1.8	1.5	2.06 ± 0.737

**Table 2:** Hematological indices in the blood of *C. idella* exposed to LC_15_ of lead for 24, 48, 72, and 96 hours.

Lead-exposed fish groups	Control				24 h LC_15_				48 h LC_15_				72 h LC_15_				96 h LC_15_			
	Fish I	Fish II	Fish III	Mean ± SD	Fish I	Fish II	Fish III	Mean ± SD	Fish I	Fish II	Fish III	Mean ± SD	Fish I	Fish II	Fish III	Mean ± SD	Fish I	Fish II	Fish III	Mean ± SD
Hemoglobin	16.1	12.6	12.2	13.6 ± 2.145	12.3	13.5	10.2	12.0 ± 1.670	15.6	7.7	6.0	9.76 ± 5.122	8.0	8.4	8.7	8.36 ± 0.351	12.1	3.9	5.0	7.0 ± 4.450
RBCs	4.05	3.16	3.07	3.4 ± 0.541	2.92	3.24	2.41	2.64 ± 0.517	4.04	2.00	1.53	2.52 ± 1.334	1.98	1.98	1.98	1.98 ± 0.0	1.96	2.92	1.53	2.13 ± 0.711
Lymphocytes	90.4	94.4	96.4	93.7 ± 3.054	94.9	96.6	96.3	95.9 ± 0.908	91.6	94.4	99.6	95 ± 4.059	97.2	97.1	96.4	96.8 ± 0.438	97.8	95.5	99.1	98.7 ± 0.88
Monocytes	8.3	4.4	3.0	5.2 ± 2.746	4.0	2.9	0.8	2.5 ± 1.625	7.2	4.4	0.2	3.93 ± 3.523	2.4	2.4	2.6	2.46 ± 0.115	1.8	0.3	0.7	0.93 ± 0.776

Hemoglobin is expressed as g·dl^−1^, RBCs are expressed as ×10^3^ *μ*l^−1^, and lymphocyte and monocyte are expressed as %.

**Table 3:** Hematological indices in the blood of *C. idella* exposed to LC_15_ of chromium for 24, 48, 72, and 96 hours.

Chromium-exposed fish groups	Control				24 h LC_15_				48 h LC_15_				72 h LC_15_				96 h LC_15_			
	Fish I	Fish II	Fish III	Mean ± SD	Fish I	Fish II	Fish III	Mean ± SD	Fish I	Fish II	Fish III	Mean ± SD	Fish I	Fish II	Fish III	Mean ± SD	Fish I	Fish II	Fish III	Mean ± SD
Hemoglobin	16.1	12.6	12.2	13.6 ± 2.145	6.5	10.9	16.9	11.4 ± 5.220	12.7	8.5	6.5	9.2 ± 3.164	7.8	9.1	8.0	8.3 ± 0.699	2.2	7.8	6.9	5.6 ± 3.007
RBCs	4.05	3.16	3.07	3.4 ± 0.541	1.41	1.99	2.96	2.12 ± 0.783	2.88	2.35	2.4	2.54 ± 0.292	2.96	2.35	1.90	2.40 ± 0.532	2.8	1.99	1.68	2.15 ± 0.578
Lymphocytes	90.4	94.4	96.4	93.7 ± 3.054	98.8	83.1	65.8	82.5 ± 16.506	97.7	95	97	96.5 ± 1.400	97	96.5	96.4	96.6 ± 0.323	97	97.8	97.7	97.5 ± 0.435
Monocytes	8.3	4.4	3.0	5.2 ± 2.746	0.7	14.2	25.1	13.3 ± 12.223	1.5	04	02	2.5 ± 1.322	2.3	3.0	3.0	2.76 ± 0.329	1.7	1.5	1.9	1.6 ± 0.2000

Hemoglobin is expressed as g·dl^−1^, RBCs are expressed as ×10^3^ *μ*l^−1^, and lymphocyte and monocyte are expressed as %.

**Table 4:** Accumulative concentration of copper, lead, and chromium in gill and muscle tissues observed after 24, 48, 72, and 96 hours of treatment (unit: mg/g).

Heavy metals	24 h		48 h		72 h		96 h	
	Gills	Muscles	Gills	Muscles	Gills	Muscles	Gills	Muscles
Copper	0.266 ± 0.029	0.038 ± 0.016	0.048 ± 0.038	0.047 ± 0.054	0.024 ± 0.007	0.021 ± 0.002	0.036 ± 0.019	0.013 ± 0.007
Lead	4.113 ± 0.831	6.697 ± 1.475	1.037 ± 0.418	3.147 ± 1.606	1.647 ± 0.136	3.253 ± 0.992	3.720 ± 3.008	3.730 ± 1.267
Chromium	0.537 ± 0.250	0.593 ± 0.393	0.240 ± 0.130	0.847 ± 0.257	0.667 ± 0.331	0.477 ± 0.162	0.937 ± 0.718	0.307 ± 0.257

**Table 5:** Representing cell and nucleus abnormalities scoring (unit: %).

Heavy metals	Treated groups	Single micronucleus	Double micronucleus	Deformed nucleus	Nuclear shift	Lobed nucleus	Irregular nucleus	Deformed cells	Microcyte cells	Vacuolated cells	Swollen cells
Copper LC_15_	Control	3	0.0	0.4	0.3	0.0	0.0	4.8	0.1	0.0	0.8
	24 h	1	0.0	0.2	0.8	0.2	0.1	7.4	1.9	0.1	1.8
	48 h	10	2	2.5	1.0	1.5	0.2	13.7	1.0	0.1	1.3
	72 h	6	2	0.9	0.4	0.8	0.3	5.4	0.6	0.0	1.5
	96 h	10	1	4.6	1.2	0.3	0.5	4.6	0.3	0.0	0.6

Chromium LC_15_	24 h	1	0.0	0.3	0.0	0.1	0.0	0.8	0.1	0.0	0.5
	48 h	2	0.0	0.5	0.1	0.0	0.5	2.2	0.2	0.2	1.1
	72 h	2	0.0	0.4	0.4	0.0	0.0	7.3	0.1	0.2	1.6
	96 h	2	0.0	1.7	0.1	0.1	0.0	3.3	0.0	0.3	4.9

Lead LC_15_	24 h	8	0.0	1.8	1.1	0.4	0.0	6.2	0.8	0.0	0.0
	48 h	7	0.0	0.9	1.9	0.4	0.6	4.2	0.3	0.1	0.3
	72 h	12	0.0	5.0	1.2	0.4	0.7	6.6	0.4	0.0	0.0
	96 h	16	0.0	2.7	1.3	0.0	1.1	1.0	0.3	0.0	2.2

## Data Availability

The data are available to readers from journal archives and Google scholars.
